# Kambo Administration and Its Association With Sudden Death: Clinical and Forensic Perspectives From a Systematic Review

**DOI:** 10.7759/cureus.77646

**Published:** 2025-01-18

**Authors:** Matteo Antonio Sacco, Saverio Gualtieri, Chara Spiliopoulou, Emmanouil I Sakelliadis, Maria Cristina Verrina, Isabella Aquila

**Affiliations:** 1 Department of Medical and Surgical Sciences, Magna Græcia University of Catanzaro, Catanzaro, ITA; 2 Department of Forensic Medicine and Toxicology, National and Kapodistrian University of Athens School of Medicine, Athens, GRC

**Keywords:** death, kambo, phyllomedusa bicolor, sapo, toxic effect

## Abstract

Kambo (Kambô) is a secretion of an amphibian traditionally used by indigenous tribes in the Amazon region. This secretion is derived from the giant monkey frog (*Phyllomedusa bicolor*) and is utilized in tribal cleansing rituals known as Kambo ceremonies. Kambo is purported to offer several benefits, both physical and psychological. Proponents of this practice claim that it can enhance physical endurance, sharpen mental clarity, and boost overall health. However, the side effects, such as tachycardia, blood pressure fluctuations, and gastrointestinal distress, highlight the need for caution. In this work, we analyzed the correlation between Kambo intake and the risk of death. A literature review demonstrated that only a few cases of death have been described to date. These reported cases emphasize the need to consider potential and concrete dangers associated with Kambo uptake, including sudden cardiac death and esophageal rupture. These episodes may be exacerbated in patients with pre-existing risk factors. Careful epidemiological and toxicological surveillance is required to timely identify these events, especially considering the difficulties related to the isolation of Kambo peptides.

## Introduction and background

The term Kambo is commonly used in scientific literature with two distinct meanings. The first refers to the tree frog *Phyllomedusa bicolor* and the secretion from its dorsal region, which is utilized in an indigenous shamanic ritual [1]. For centuries, indigenous groups have used Kambo as a medicinal tool to "strengthen the body's natural defenses," ward off "panem" (misfortune), and as part of rituals meant to improve hunting success. After the colonization of Brazil’s Acre state, rubber tappers began adopting this practice, and the ritual is now common in various Brazilian regions, including large cities. The administration of Kambo is not confined to a purely spiritual context; it also has a more pragmatic aspect, with regular citizens using it for healing purposes. The secretion of the frog is collected on a wooden tool that absorbs it for ritual use [2].

Over the past two decades, Kambo has made its way to urban centers in Brazil and worldwide, marketed as a "detox" treatment. From its Amazonian origins to its role in Brazilian syncretic religions like Santo Daime and União do Vegetal, it has eventually gained popularity in Western healing circles, especially among cancer patients who find no benefit from conventional medicine [3]. This substance, native to South America, has spread to other parts of the world, including Europe and the United States [4].

Compared to most amphibians, *Phyllomedusa bicolor *has a wide distribution, inhabiting tropical forests in the Amazon regions of Bolivia, Peru, French Guiana, Colombia, Venezuela, Guyana, Suriname, and Ecuador [1].

Kambo has often been linked to the use of serotonergic psychedelics like ayahuasca, with induced vomiting seen as a cleansing of impurities from the body [5]. In Brazil, as Kambo consumption has risen in urban centers, criticism has emerged, particularly from indigenous groups, academics, and media. This criticism centers around the cultural appropriation of indigenous knowledge, the extraction of frog secretions, the transmission of wisdom, the cost of the ritual, and the mystification surrounding the frog’s origin [6]. It is important to note that the sale and marketing of Kambo have been illegal in Brazil since 2016.

In Australia, the deaths of two individuals after undergoing Kambo rituals have prompted forensic and toxicological investigations into the substance. As a result, it was classified as a poison by the Therapeutic Goods Administration in 2021, placed in Annex 10: "a substance with such a health hazard that its sale, supply, and use are prohibited."

Elsewhere in the world, however, Kambo remains largely unknown, and many institutions do not yet recognize it as a poison. This lack of awareness presents a major medicolegal challenge, especially as traditional therapies are adopted in the West. The route of administration and failure to adhere to standard practices can lead to fatalities [6].

Typically, the substance is administered transdermally through the application of a hot stick by a shaman, creating small circular burns ("dots") on the skin [5]. These application sites are usually on the upper limbs in men and lower limbs in women.

The frog *Phyllomedusa bicolor *releases narcotic substances from three types of epidermal glands, classified by the chemical and physical nature of their secretion: serous, mucous, and lipid. The biologically active peptides used in rituals are secreted from the serous glands on the frog’s back, serving a passive anti-predatory defense function. These glands produce propeptides, which are stored in vesicles and mature through the removal of the signal peptide and C-terminal portion. Once matured, the peptides rearrange into granules within the internal periplasmic space, which contains proteins with clinical and medical significance, such as dermaseptin, dermatoxin, deltorfine, phyllocareuline, and sauvagina [7-9].

High concentrations of these peptides are present in the frog’s secretions, which are quickly absorbed through damaged skin, inducing neuroexcitation, relaxation of smooth vascular muscle, and opiate-like effects that last about 15 minutes. However, other effects include neuropsychiatric symptoms like confusion, memory loss, lethargy, convulsions, psychosis, inappropriate secretion of antidiuretic hormones (SIADHs), and damage to the kidneys, pancreas, and liver (including hepatitis), primarily due to the action of deltorfin proteins, which are high-affinity δ-opioid receptor agonists.

Kambo is also known to be traditionally used for inducing abortions, and thus pregnant women should avoid participating in this ritual. Excessive use or treatment of children with lower body mass should also be avoided, as the mass-to-dose ratio may be more significant in these cases [2]. Psychiatric effects and altered states of consciousness are induced by hyperthermia and hyponatremia, often misinterpreted by participants as "astral travel," instead of being recognized as potentially fatal conditions [10,11].

These adverse effects are particularly prevalent in areas where non-traditional practitioners operate. One challenge in evaluating the risks of these rituals is the unknown number of practitioners. In addition, the uncontrolled nature of Internet sales exacerbates the situation. Inexperienced practitioners may inadvertently use skin secretions from other amphibian species, such as the cane toad *Rhinella marina* [4,11].

This study aims to demonstrate that improper handling of the "toad vaccine" can lead to significant harm or even death. With the spread of this practice through media globalization and the Internet, the number of cases and consumers is likely to increase. Therefore, policymakers in various countries should take measures to prevent the spread of this dangerous substance worldwide. 

## Review

Materials and methods

A systematic literature search was conducted following the PRISMA (Preferred Reporting Items for Systematic Reviews and Meta-Analyses) guidelines to ensure comprehensiveness and transparency [12]. Relevant studies were identified through a thorough search of the PubMed and Scopus databases. No time or regional limitations were applied, and only articles written in English or Spanish were included. The primary search term used was "Kambo," and references from selected articles were reviewed for further relevant papers. Studies citing these articles were also searched in PubMed and Scopus, and relevant papers were accessed. The literature search began in July 2024, coinciding with the project’s inception, and continued through September 2024. The last access date was September 30, 2024.

This review includes cases where the Kambo administration resulted in death and cases where subjects survived after appropriate medical intervention. The extracted data includes authorship, publication year, study region, study aims, results, toxicological investigations, associated symptoms, and details of the medical interventions provided. By including survival cases, this review aims to provide a comprehensive overview of both fatal and non-fatal outcomes of Kambo use.

Due to the qualitative nature of this review and significant variations in study designs, no meta-analysis or statistical analysis was conducted. The findings are presented descriptively, emphasizing patterns in clinical outcomes, toxicological findings, and the impact of timely medical intervention on survival rates.

Results

A total of 123 articles were retrieved from the PubMed and Scopus databases. After excluding irrelevant papers and duplicates, 18 articles were reviewed in full text. Following the expansion of the inclusion criteria to encompass cases where subjects survived after appropriate medical intervention, a final count of nine relevant articles met the established criteria. These included both fatal cases and survival cases, providing a broader perspective on the clinical outcomes of Kambo administration.

A quality assessment was conducted using the Joanna Briggs Institute (JBI) critical appraisal tools [13-14], and all included articles met the minimum quality standards for inclusion. The analysis highlights the diversity in reported outcomes, ranging from severe complications requiring extensive medical intervention to fatal cases, underscoring the spectrum of risks associated with Kambo use.

**Figure 1:**
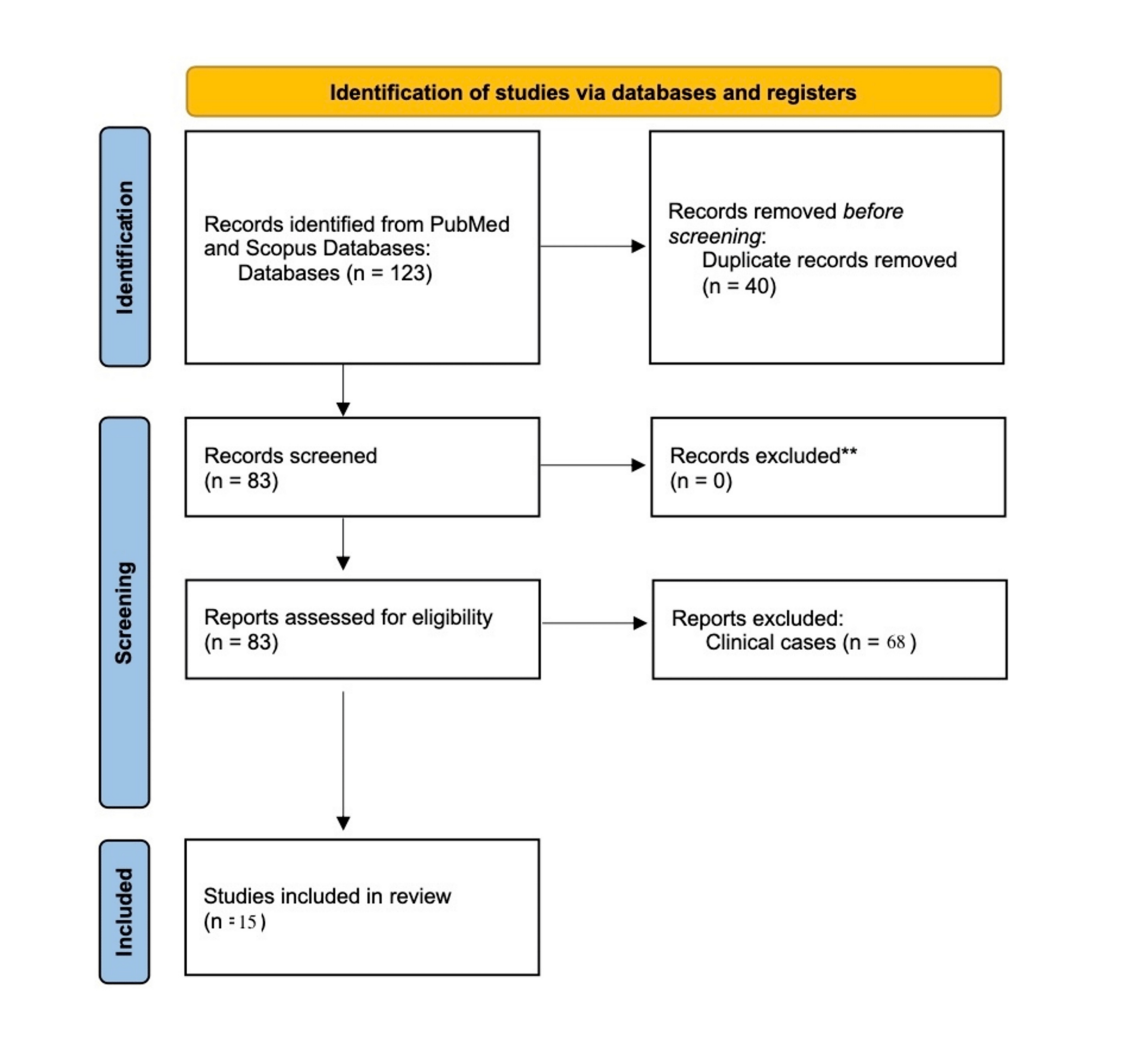
Preferred Reporting Items for Systematic Reviews and Meta-Analyses (PRISMA) flow diagram

The included papers are summarized in a PRISMA flow diagram (Figure 1) and the results are presented in Table 1, listing study details such as authorship, year of publication, study region, aim, results, and toxicological findings [11-27].

**Table 1:** Results of intoxication cases due to Kambo SIADH: syndrome of inappropriate antidiuretic hormone secretion

Authors	Year	Region	Aim	Subject of the article	Toxicological investigation performed
Aquila I et al. [15]	2018	Italy	Case report	Sudden death, 30 minutes after transdermal Kambo administration, of a 42-year-old Italian man.	Deltorphine A in a blood sample (5.00 μg/mL). No cannabinoids, cocaine, benzodiazepines, or ethanol. Deltorphine A, phyllocaerulein, and phyllokinin found in sticks.
Byard RW et al. [11]	2020	Australia	Editorial	A female in New South Wales died during a Kambo ceremony. Cause of death under coronial investigation.	Not reported. Highlighted issues with toxicological methodology for organic toxin detection.
Robalino Gonzaga ES et al. [16]	2020	United States	Case report	A female after Kambo administration exhibited severe vomiting, leading to esophageal rupture, tension pneumothorax, and septic shock.	Not reported.
Silva FVAD et al. [2]	2019	Brazil	Letter to the editor	A 52-year-old male died following a Kambo application.	Not reported.
Darlington et al. [20]	2023	Australia	Case report	Boerhaave syndrome in an otherwise-healthy male following Kambo use.	Not reported.
Peleg Hasson et al. [3]	2021	Europe	Case report	Systemic inflammatory response syndrome (SIRS) mimicking disease progression in a patient with cholangiocarcinoma after Kambo use.	Not reported.
Schmidt et al. [5]	2020	Global	Retrospective study	Acute and subacute psychoactive effects of Kambo use in urban healing circles, revealing mild to moderate acute effects and positive persisting psychological effects.	Not reported.
Alamos et al. [22]	2020	Chile	Case report	Severe neurologic effects, including hallucinations and seizures, in a 41-year-old woman treated with Kambo for depression. She required mechanical ventilation, developed rhabdomyolysis and renal failure, and fully recovered within seven days.	Not reported.
De la Vega et al. [21]	2020	Global	Case report	Dermatomyositis in a 33-year-old female following periodic use of Kambo toxin. Symptoms included myalgia, muscle weakness, and elevated muscle enzymes. Diagnosis confirmed using classification criteria. Full recovery with prednisone treatment in eighr weeks.	Not reported.
Campodónico et al. [23]	2019	Chile	Case report	Severe hyponatremia (120 mEq/L) and rhabdomyolysis in a 41-year-old female after Kambo use during a healing ritual. Symptoms included tonic-clonic seizures, psychomotor agitation, and SIADH. Full recovery after correction of hyponatremia and management of rhabdomyolysis.	Toxicological screening did not identify other substances.
Roy et al. [24]	2018	United States	Case report	A 33-year-old female developed psychosis after frequent Kambo rituals, presenting with paranoia, anxiety, bizarre delusions, and panic attacks. Symptoms improved with risperidone treatment during a nine-day hospitalization.	Extensive medical workup was unremarkable.
Kumachev et al. [25]	2018	Canada	Case report	A 32-year-old female presented with nausea, vomiting, and abdominal discomfort after a Kambo ritual. Treated with IV fluids, ondansetron, and naloxone, she fully recovered within one hour.	Positive for cannabinoids on toxicology screen. The remainder of the laboratory workup was unremarkable.
Li K et al. [26]	2018	United States	Case report	A patient presented 22 hours after a Kambo cleanse with prolonged vomiting, flushing, facial swelling, altered mental status, and agitation requiring chemical restraints. Symptoms resolved with symptomatic treatment.	Not reported.
Pogorzelska et al. [18]	2017	Europe	Case report	A 34-year-old male developed toxic hepatitis associated with the Kambo ritual, presenting with elevated liver enzymes, jaundice, and abdominal pain. Symptoms resolved after 10 days of symptomatic treatment.	Extensive testing excluded infections and other causes of liver damage.
Leban et al. [27]	2016	Slovenia	Case report	A 44-year-old female developed SIADH and acute symptomatic hyponatremia after a Kambo ritual, presenting with confusion, lethargy, muscle weakness, spasms, and seizures. Symptoms improved with sodium chloride and water restriction.	Plasma and urine osmolality tests confirmed SIADH.

Kambo, as part of a traditional Amazonian cleansing ritual, is known for its severe short-term side effects, which often appear minutes after administration. These include intense nausea, vomiting, diarrhea, and profuse sweating. The gastrointestinal distress associated with Kambo is typically described as extreme, with some individuals experiencing significant discomfort. Physical responses such as tachycardia and edema further amplify the distress, making the experience particularly overwhelming [1].

The effects of Kambo on the cardiovascular system are notably significant and have been documented in various studies. Kambo administration influences both heart rhythm and blood pressure, frequently resulting in hypotension and tachycardia. These cardiovascular responses can be attributed to peptides such as phyllokinin and phyllomedusin, which induce vasodilation and hypotension. Beyond the immediate and intense effects of Kambo, potential long-term health complications also exist, warranting serious consideration. Reports have associated Kambo use with severe health outcomes such as seizures, liver failure, and even heart attacks, which may manifest as sudden cardiac death [15]. These serious conditions underscore the potential for Kambo to cause lasting damage, particularly in individuals with pre-existing health conditions.

In addition to acute reactions, the potential long-term health consequences of Kambo use should be carefully evaluated. Reports have linked Kambo use to complications like seizures, liver failure, and heart attacks, sometimes resulting in sudden cardiac death. These conditions illustrate the profound and lasting damage Kambo can inflict, particularly on individuals with underlying health issues [17-18]. The lack of scientific evidence supporting Kambo’s safety and efficacy underscores the necessity for caution. Documented cases of acute intoxication leading to life-threatening effects emphasize that the risks extend beyond immediate discomfort.

Several factors contribute to the fatalities associated with Kambo use, adding complexity to its risks. In this review, both fatal and non-fatal cases have been included. Some of these cases, summarized in Table 1, highlight not only the adverse outcomes but also instances where timely medical intervention proved effective in managing severe symptoms and preventing mortality. These survival cases provide valuable insights into the critical role of appropriate medical care in mitigating the risks of Kambo administration.

Pre-screening participants for pre-existing medical conditions is critical to ensuring their safety during Kambo rituals. Individuals with underlying health conditions may be particularly susceptible to Kambo’s effects, including tachycardia and hypotension. Furthermore, the inclusion of survival cases underscores the importance of developing protocols for risk assessment and participant screening before administering Kambo.

From a forensic toxicological perspective, isolating Kambo peptides remains challenging due to multiple factors. The primary difficulty lies in the complexity of the peptide mixture present in Kambo secretions, which includes compounds like phyllokinin, phyllomedusin, and deltorphin A. These peptides often degrade rapidly in biological samples, making their detection and quantification problematic. Furthermore, the lack of standardized analytical methods and peptide-specific standards complicates the application of routine toxicological protocols. Advanced techniques, such as liquid chromatography coupled with mass spectrometry (LC-MS), are required to accurately identify and measure these compounds. However, these methods are not universally available, particularly in regions where Kambo use is more prevalent. This lack of resources limits the ability to perform consistent toxicological investigations. Moreover, the absence of a comprehensive database for Kambo peptides and their metabolites further hinders reliable interpretation of toxicological findings. Consequently, forensic toxicologists must rely on circumstantial evidence and case-specific findings to draw conclusions about suspected Kambo-related intoxications. Except for the work by Aquila et al. (2018), most studies encounter technical difficulties due to the lack of appropriate peptide standards [15]. Consequently, thorough investigations at the scene and the collection of circumstantial evidence are essential in suspected Kambo-related intoxications. Such cases should be promptly reported to health authorities to support epidemiological data collection.

Timely medical intervention has been shown to significantly reduce mortality rates, as prompt treatment can address acute cardiovascular effects such as tachycardia, tachypnea, and hypotension [19-20]. Survival cases demonstrate that early recognition of symptoms, coupled with immediate medical care, can dramatically improve outcomes. Healthcare professionals should remain vigilant for symptoms such as facial swelling, sweating, and fainting, which occur in a significant proportion of cases.

Legal and ethical issues surrounding Kambo use are multifaceted. In the United States, Kambo is not explicitly banned, but its use is strictly regulated. Ethical concerns also emerge due to the lack of adequate medical supervision during administration, which presents substantial risks. Documented cases of harm and death emphasize the need for regulatory measures to protect users [21].

The widespread availability of Kambo on the Internet poses another pressing concern, contributing to an uncontrolled increase in fatalities. Forensic pathologists play a crucial role in identifying characteristic external signs, such as burns, which enable further investigation into Kambo administration. The inclusion of survival cases in this review highlights the importance of raising awareness about the risks of Kambo use and the need for standardized guidelines to ensure participant safety. Kambo’s growing popularity beyond traditional contexts necessitates a careful balance between preserving its cultural significance and minimizing the associated risks.

## Conclusions

Kambo, a traditional ritual from the Amazon, has garnered global attention for its claimed therapeutic effects, but it also presents significant health risks. This review highlights both the immediate and long-term hazards linked to Kambo use, including cardiovascular issues, gastrointestinal distress, and the potential for fatal consequences, particularly in individuals with underlying health conditions. The reported cases of fatalities and severe health complications underscore the urgent need for rigorous screening protocols, especially for those with pre-existing medical conditions.

The challenges associated with the forensic identification of Kambo peptides, coupled with the lack of conclusive scientific data, stress the importance of further comprehensive research to determine its safety and efficacy. Public health policies must address the unregulated sale and use of Kambo, especially through online channels, to prevent misuse and minimize harm. Furthermore, the ethical concerns surrounding cultural appropriation and the absence of medical oversight during Kambo rituals call for the implementation of appropriate regulatory frameworks and culturally respectful practices.
